# Association of extracellular dNTP utilization with a GmPAP1-like protein identified in cell wall proteomic analysis of soybean roots

**DOI:** 10.1093/jxb/erx441

**Published:** 2018-01-05

**Authors:** Weiwei Wu, Yan Lin, Pandao Liu, Qianqian Chen, Jiang Tian, Cuiyue Liang

**Affiliations:** 1 Root Biology Center, State Key Laboratory for Conservation and Utilization of Subtropical Agro-bioresources, South China Agricultural University, Guangzhou, P. R. China; 2 Institute of Tropical Crop Genetic Resources, Chinese Academy of Tropical Agriculture Sciences, Hainan, P. R. China

**Keywords:** Cell wall proteins, soybean, phosphorus deficiency, phosphorus utilization, proteomics, purple acid phosphatase

## Abstract

Plant root cell walls are dynamic systems that serve as the first plant compartment responsive to soil conditions, such as phosphorus (P) deficiency. To date, evidence for the regulation of root cell wall proteins (CWPs) by P deficiency remains sparse. In order to gain a better understanding of the roles played by CWPs in the roots of soybean (*Glycine max*) in adaptation to P deficiency, we conducted an iTRAQ (isobaric tag for relative and absolute quantitation) proteomic analysis. A total of 53 CWPs with differential accumulation in response to P deficiency were identified. Subsequent qRT-PCR analysis correlated the accumulation of 21 of the 27 up-regulated proteins, and eight of the 26 down-regulated proteins with corresponding gene expression patterns in response to P deficiency. One up-regulated CWP, purple acid phosphatase 1-like (GmPAP1-like), was functionally characterized. *Phaseolus vulgaris* transgenic hairy roots overexpressing *GmPAP1-like* displayed an increase in root-associated acid phosphatase activity. In addition, relative growth and P content were significantly enhanced in *GmPAP1-like* overexpressing lines compared to control lines when deoxy-ribonucleotide triphosphate (dNTP) was applied as the sole external P source. Taken together, the results suggest that the modulation of CWPs may regulate complex changes in the root system in response to P deficiency, and that the cell wall-localized GmPAP1-like protein is involved in extracellular dNTP utilization in soybean.

## Teaser

A cell wall-localized GmPAP1-like protein is up-regulated by Pi starvation, and is involved in extracellular dNTP utilization in soybean.

## Introduction

Phosphorus (P) is a critical macronutrient in plants that not only serves as a major structural component, but also acts directly and indirectly in multiple metabolic processes, such as membrane and nucleotide synthesis, photosynthesis, energy transmission, and signal transduction (Raghothama, 1999; Vance *et al.*, 2003; Plaxton and Lambers, 2015). Available phosphate (Pi) commonly limits crop production on arable lands worldwide, especially on acid soils (von Uexküll and Mutert 1995; Vance *et al.* 2003). In nature, plants have developed a set of strategies to enhance P acquisition and utilization efficiency in P-limited soils (Liang *et al.*, 2014). Multiple adaptive strategies to low-P stress have been well documented in roots, including modification of root architecture and morphology, increased root exudation of organic acids and purple acid phosphatase (PAP), and enhanced symbiotic association with arbuscular mycorrhiza fungi (Chiou and Lin, 2011; Wu *et al.*, 2013; Liang *et al.*, 2014; Plaxton and Lambers, 2015).

In plant roots, cell walls are directly connected to the rhizosphere environment, and are thus the first compartment to perceive and transmit extra- and intercellular signals in many pathways, and to do this they rely on the enzymatic activities of cell wall proteins (CWPs) (Jamet *et al.*, 2008; Zhu *et al.*, 2012, 2016; Hoehenwarter *et al.*, 2016). CWPs only account for approximately 10% of cell wall dry mass, and yet they include several hundred proteins acting across wide-ranging functions (Fry, 1988; Cosgrove, 1997; Borderies *et al.*, 2003; Lee *et al.*, 2004; Bayer *et al.*, 2006). CWPs can be divided into three categories according to their binding properties, namely labile proteins that exhibit little or no interaction with other cell wall components, weakly bound proteins that are extractable with salt solutions, and strongly bound proteins that are only released by intensive extraction treatments (Jamet *et al.*, 2006, 2008; Komatsu and Yanagawa, 2013). Although relatively minor in quantity, CWPs are critical for the maintenance of biological functionality in the plant extracellular matrix (Somerville *et al.*, 2004; Bayer *et al.*, 2006; Zhu *et al.*, 2006). Using a variety of proteomics techniques, CWPs have been identified in a range of plant species, including *Arabidopsis thaliana* (Bayer *et al.*, 2006; Minic *et al.*, 2007), *Medicago sativa* (Soares *et al.*, 2007), chickpea (*Cicer arietinum*) (Bhushan *et al.*, 2006), maize (*Zea mays*) (Zhu *et al.*, 2006, 2007), rice (*Oryza sativa*) (Jung *et al.*, 2008), and sugar cane (*Saccharum officinarum*) (Calderan-Rodrigues *et al.*, 2014). Despite this, there is a scarcity of proteomics data available for CWP responses to mineral nutrient deficiencies, particularly P deficiency.

In recent years, functional analysis of several Pi starvation-responsive and cell wall-localized proteins has shed light on vital roles of CWPs involved in plant adaptation to P deficiency. For example, nine β-expansin members were identified in soybean (Li *et al.*, 2014), one of which, *GmEXPB2*, is up-regulated by Pi starvation and appears to play an important role in mediating root growth, suggesting that alteration of cell wall structure is critical for plant adaptation to P deficiency (Guo *et al.*, 2011). Additionally, two cell wall-localized purple acid phosphatases (PAPs), NtPAP12 in tobacco and AtPAP25 in Arabidopsis, are suggested to be involved in cell wall synthesis (Kaida *et al.*, 2008, 2009, 2010; Del Vecchio *et al.*, 2014). Beyond mediation of cell wall biosynthesis, extracellular and cell wall-localized PAPs have also been found to participate in extracellular P scavenging and recycling (Tran *et al.*, 2010*a*, 2010*b*; Tian and Liao, 2015). In Arabidopsis, three secreted PAPs (AtPAP10, AtPAP12, and AtPAP26) account for the bulk of the enhanced secreted acid phosphatase activity observed with P deficiency (Hurley *et al.*, 2010; Wang *et al.*, 2011, 2014; Robinson *et al.*, 2012). These secreted PAPs possess high activities against a wide range of organic phosphomonoesters, and are suggested to participate in extracellular organic P [e.g. ATP, ADP, and dNTP (deoxy-ribonucleotide triphosphate)] utilization (Hurley *et al.*, 2010; Tran *et al.*, 2010*b*; Wang *et al.*, 2011, 2014; Robinson *et al.*, 2012). Similar results have also been observed for extracellular PAPs in other plants, including bean (Liang *et al.*, 2010, 2012), rice (Lu *et al.*, 2016), and *Stylosanthes* (Liu *et al.*, 2016). However, the functions of cell wall-localized PAPs remain largely unknown in soybean and many other crops.

Soybean is an important legume crop that is a valuable source of protein and vegetable oil for human consumption (Herridge *et al.*, 2008). Low Pi availability inhibits soybean growth and production on many soils (Zhao *et al.*, 2004; Ao *et al.*, 2010; Wang *et al.*, 2010). Although a number of adaptive strategies to Pi starvation have been observed in soybean, along with series of Pi starvation-responsive genes (Tian *et al.*, 2003; Zhao *et al.*, 2004; Ao *et al.*, 2010; Wang *et al.*, 2010; Guo *et al.*, 2011; Li *et al.*, 2012; Qin *et al.*, 2012; Yao *et al.*, 2014), a proteomic-level characterization of soybean root CWPs in low-Pi conditions has yet to be reported. In this study, an iTRAQ (isobaric tag for relative and absolute quantitation) proteomics assay was conducted with soybean roots to identify water-soluble and weakly bound CWPs that are responsive to Pi starvation. The CWPs that were identified as having differential accumulation were then functionally classified. Finally, a cell wall-localized PAP, *GmPAP1-like*, was tested for differential expression and participation in extracellular organic-P utilization.

## Materials and methods

### Plant material and growth conditions

For low-Pi experiments, the soybean (*Glycine max*) genotype YC03-3 was grown in hydroponic culture. Seeds were germinated in paper rolls moistened with half-strength nutrient solution after surface-sterilization with 10% (v/v) H_2_O_2_ as previously described (Liang *et al.*, 2013). The full-strength nutrient solution without phosphate was composed of (in mM): 1.5 KNO_3_, 1.2 Ca(NO_3_)_2_, 0.4 NHNO_3_, 0.025 MgCl_2_, 0.5 MgSO_4_, 0.3 K_2_SO_4_, 0.3 (HN_4_)_2_SO_4_, 0.0015 MnSO_4_, 0.0015 ZnSO_4_, 0.0005 CuSO_4_, 0.00015 (NH_4_)_6_Mo_7_O_24_, 0.0025 H_3_BO_3_, and 0.04 Fe-Na-EDTA. Five days after seed germination, uniform seedlings were transplanted to full-strength nutrient solution containing 5 µM (–P) or 250 µM (+P) KH_2_PO_4_. The nutrient solution was continuously aerated and replaced every week. Dry weight and P content were determined 10 d after transplantation for both shoots and roots, together with root length, average diameter, and surface area. Roots were also harvested for extraction of water-soluble and weakly bound CWPs, assays of acid phosphatase (APase) activity, and analysis of gene expression.

For the gene temporal expression assay, soybean seeds were surface-sterilized and germinated as described above. After germination, uniform seedlings were transplanted to –P or +P nutrient solutions. Samples of roots were harvested for RNA extraction at three growth stages, namely seedling (7 d after transplanting), flowering (31 d after transplanting), and maturity (51 d after transplanting). All experiments had four biological replicates.

To assay genotypic variation of gene expression, nine soybean genotypes, contrasting in P-use efficiency were selected: YC03-3, NH89, NH112, ZDD06270, ZDD04072, ZDD12944, ZDD04044, ZDD20323, and ZDD16817 (Zhao *et al.*, 2004). After seed germination, uniform seedlings were grown in the full-strength nutrient solution containing 5 µM (–P) or 250 µM (+P) KH_2_PO_4_ as described above. After 10 d, roots were separately harvested for gene expression analysis. All experiments had four biological replicates.

### Measurement of plant P content

Plant P content was measured as previously described, with modifications (Murphy and Riley, 1962; Qin *et al.*, 2012). Briefly, about 0.1 g of dry soybean plants or transgenic bean (*Phaseolus vulgaris*) hairy root samples were ground into powder and digested by boiling with H_2_SO_4_ and H_2_O_2_. Each supernatant was then transferred to a volumetric flask with the volume adjusted to 100 ml using deionized water. Phosphorus content in the solution was then measured using a Continuous Flow Analytical System (Skalar, Holland) according to the user manual.

### Identification of water-soluble and weakly bound cell wall proteins from soybean roots

Proteins weakly bound to the cell wall were extracted from both P-deficient and P-sufficient soybean roots as previously described (Feiz *et al.*, 2006). Briefly, 4 g of soybean roots from each of the four biological replicates were pooled and ground into a homogeneous slurry in a cold-room. The mixture was then centrifuged for 15 min at 1000 *g* and 4 °C to separate cell walls from soluble cytoplasmic fluid. Pellets were washed with 5 mM acetate buffer, pH 4.6, followed by 0.6 M and then 1 M sucrose, before a final wash with 3 l of 5 mM acetate buffer, pH 4.6. The resulting cell wall fraction was ground into powder in liquid nitrogen and lyophilized prior to protein extraction. Weakly bound proteins were obtained by two extractions with 5 mM acetate buffer containing 0.2 M CaCl_2_, followed by two extractions with 5 mM acetate buffer containing 2 M LiCl. The products were pooled, desalted using Econo-Pac^®^ 10DG desalting columns (BIO-RAD, USA), lyophilized, and used for the iTRAQ proteomics assay. Briefly, proteins were digested with Trypsin Gold (Promega, Madison, WI, USA) with a ratio of protein:trypsin of 30:1 at 37 °C for 16 h. After digestion, the peptides were dried by vacuum centrifugation. The peptides were reconstituted in 0.5M TEAB (triethyl ammonium bicarbonate) and processed according to the manufacture’s protocol for 8-plex iTRAQ reagent (Applied Biosystems). Briefly, one unit of iTRAQ reagent was thawed and reconstituted in 24 μl isopropanol. Samples were labeled with the iTRAQ tags as follows: protein extracted from P-sufficient roots was labeled with 117-tag and protein extracted from P-deficient roots was labeled with 114-tag following the manufacturer’s recommended protocol (iTRAQ® Reagents, USA), and samples were analysed by nanoLC-MS/MS after going through a chromatography separation. Data acquisition was performed with a TripleTOF 5600 System (AB SCIEX, Concord, Canada) fitted with a Nanospray III source (AB SCIEX, Concord, Canada) and a pulled-quartz tip as the emitter (New Objectives, Woburn, USA). Raw data files acquired from the TripleTOF 5600 were converted into MGF files using 5600 msconverter and the MGF files were searched (see Dataset 1 available at Dryad Digital Repository http://dx.doi.org/10.5061/dryad.6t1f5). Protein identification was performed by using the Mascot search engine (Matrix Science, version 2.3.02, UK). For protein identification, the charge states of peptides were set to +2 and +3. Specifically, an automatic decoy database search was performed in Mascot by choosing the decoy checkbox in which a random sequence of database was generated and tested for raw spectra as well as the real database. To reduce the probability of false peptide identification, only peptides at the 95% confidence interval according to a Mascot probability analysis were counted as identified. In addition, each confidently identified protein had at least one unique peptide. For protein quantization, it was required that a protein contained at least two unique spectra. The quantitative protein ratios were weighted and normalized using the median ratio in Mascot. Proteins scoring higher than 60, ratios with *P*-values <0.05 (expectation value), and fold-changes >1.5 were considered as significant.

Secreted proteins with signaling peptides were predicted using the Signal-P V3 program (http://www.cbs.dtu.dk/services/TargetP/) as previously described (Bendtsen *et al.*, 2004; Zhu *et al.*, 2006). In addition, the Secretome P 1.0 Server was also used for the prediction of potential CWPs secreted through a non-classic secretion pathway (http://www.cbs.dtu.dk/services/SecretomeP-1.0/) (Bendtsen *et al.*, 2004; Zhu *et al.*, 2006).

### RNA extraction and quantitative real-time PCR

Total RNA was extracted from soybean roots and transgenic bean hairy roots using the RNA-solve reagent (OMEGA bio-tek, USA). Total RNA was treated with RNase-free DNase I (Invitrogen, USA) to remove genomic DNA prior the production of reverse-transcripts using MMLV-reverse transcriptase (Promega, USA) following the instructions in the manuals. Synthesized first-strand cDNA was used for SYBR Green-monitored qRT-PCR analysis on a Rotor-Gene 3000 real-time PCR system (Corbett Research, Australia). qRT-PCR primer pairs (Table S3 at Dryad) were designed according to the deduced cDNA sequences of the differentially expressed CWPs. Primer pairs for *GmEF-1a* (accession no. X56856; 5′-TGCAAAGGAGGCTGCTAACT-3′ and 5′-CAGCATCACCGTTCTTCAAA-3′) and *PvEF-1a* (accession no. PvTC3216; 5′-TGAACCACCCTGGTCAGATT-3′ and 5′-TCCAGCATCACCATTCTTCA-3′) were used as housekeeping-gene controls to normalize the expression of the corresponding genes in soybean and the expression of *GmPAP1-like* in transgenic bean hairy roots, respectively.

### Assay of APase activity

Internal APase activity was determined using the extracts from soybean roots. Root proteins were extracted and incubated in 45 mM sodium acetate buffer containing 1 mM ρ-nitrophenylphosphate (ρ-NPP) at 35 ℃ for 15 min as previously descried (Liang *et al.*, 2010). The amount of released nitrophenol from ρ-NPP was then quantified by measuring absorbance at 405 nm (A_405_).

Quantitative and staining analyses of root-associated APase activity were conducted following published protocols (Liu *et al.*, 2016). Briefly, for quantitative analysis, transgenic bean hairy roots were incubated for 20 min in 45 mM Na-acetate buffer containing 2 mM ρ-NPP (pH 5.0). The reaction was terminated by adding 1 ml of 1 M NaOH prior to measuring A_405_. Root-associated APase activity was expressed as micromoles of ρ-NPP hydrolysed per minute per gram of roots.

For root-associated APase activity staining, soybean roots and transgenic bean hairy roots were placed on solid Murashige and Skoog (MS) medium and covered with 0.5% (w/v) agar containing 0.02% (w/v) of 5-bromo-4-chloro-3-indolyl-phosphate (BCIP; Sigma, USA). After 2 h of incubation at 25 °C, root-associated APase activity was indicated by the intensity of blue color on root surfaces. Images were then captured by a single-lens reflex camera (Canon, Japan).

### Isolation and subcellular localization of GmPAP1-like protein

Full-length cDNA of soybean purple acid phosphatase 1-like, *GmPAP1-like*, was amplified and sequenced. The deduced amino acid sequence of the GmPAP1-like protein together with PAP homologues from other plant species were used for phylogenetic tree analysis using the neighbor-joining method with 1000 bootstrap replicates in the MEGA 5 program.

The primers, 5′-CTCTAGCGCTACCGGTATGATGATGA GTGGGATGG-3′ and 5′-CATGGTGGCGACCGGTGCAGAT GCTAGTGTTGTAGCTGGAC-3′ were used to amplify *GmPAP1-like*. The PCR product was subsequently cloned into the *pEGAD* vector to produce a *GmPAP1-like-GFP* construct. The construct was then transformed into bean hairy roots following published methods (Liang *et al.*, 2010). The *pEGAD* empty vector with *GFP* expression driven by a *CaMV 35S* promoter was used as a control. Cell walls were indicated by propidium iodide (PI) staining (Liang *et al.*, 2010). Green fluorescence derived from GFP and red fluorescence derived from PI were observed by confocal scanning microscopy at 488 nm and 636 nm, respectively (LSM780, Zeiss, Germany).

### Functional characterization of GmPAP1-like in bean hairy roots

The full length of *GmPAP1-like* was then subcloned into the modified *pEGAD* vector to produce a *35S::GmPAP1-like* over-expression construct. Plasmids of the *GmPAP1-like* over-expression construct and the empty vector were transformed into bean hairy roots as previously described (Liang *et al.*, 2010). Transgenic hairy roots verified by qRT-PCR analysis were then used to analyse root-associated APase activity and the utilization of extracellular dNTPs by GmPAP1-like. The dNTP utilization experiments were conducted as described previously (Liang *et al.*, 2012; Liu *et al.*, 2016) with minor modifications. Briefly, uniform transgenic bean hairy roots were supplied with either 1.2 mM KH_2_PO_4_ or 0.4 mM dNTP in solid MS medium as the sole external P source. Fresh weight and P content of hairy roots were determined 14 d after initiation of the P treatments. Three independent lines were tested with each treatment using four biological replicates.

### Statistical analysis

All data were analysed by Student’s *t*-test using SPSS software (SPSS Institute, USA).

## Results

### Effects of Pi starvation on soybean biomass and P content

**Fig. 1.**
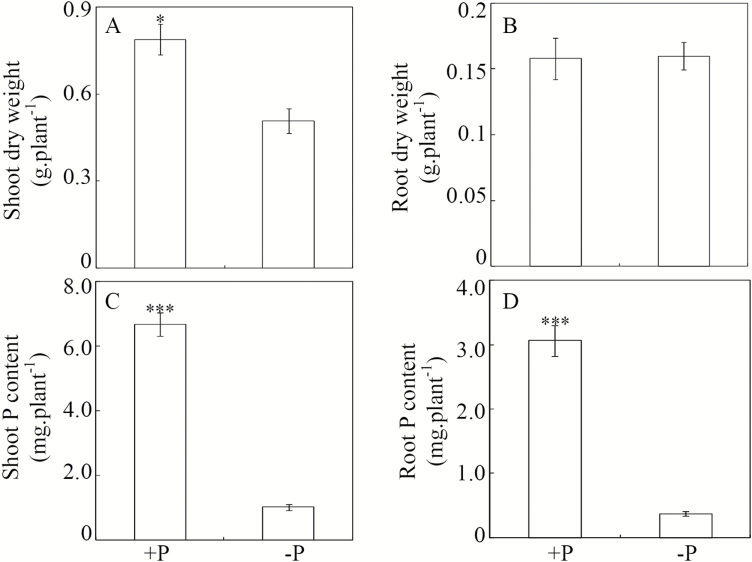
Effects of P availability on soybean dry weight and P content. Soybean seedlings were treated with +P (250 μM KH_2_PO_4_) or –P (5 μM KH_2_PO_4_). Data are means of four replicates ±SE. Asterisks indicate significant differences between the two P treatments: *P<0.05; ***P<0.001.

Low P availability significantly affected soybean growth in hydroponic culture, as reflected by decreases in plant dry weight and total P content relative to plants grown in +P solutions (Fig. 1). P-deficient plants exhibited a 35% reduction in shoot dry weight compared with the P-sufficient plants (Fig. 1A). In contrast, no significant difference in root dry weight was observed between the two P treatments (Fig. 1B). Total P content was significantly lower in plants grown under low-P conditions than in those grown under P-sufficient conditions. Total shoot and root P contents were 6.6 and 8.2 times lower, respectively, in P-deficient plants than in P-sufficient plants (Fig. 1C, D).

### Effects of Pi starvation on soybean root morphology

**Fig. 2.**
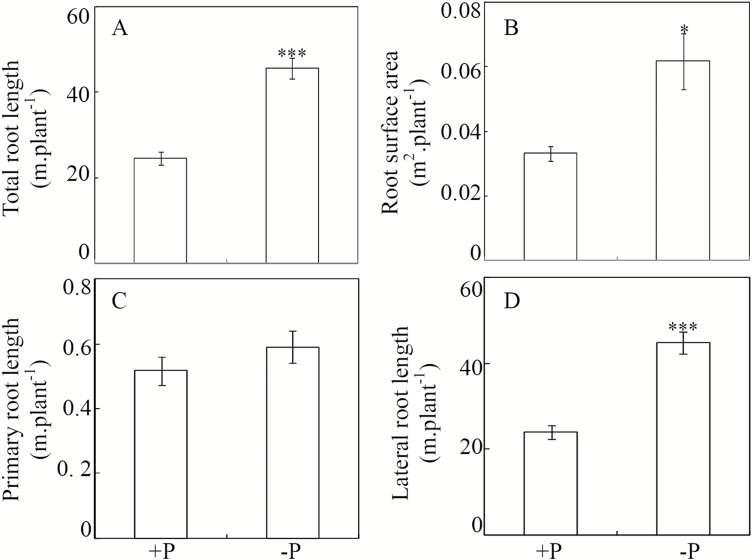
Effects of P availability on soybean root growth. Soybean seedlings were treated with +P (250 μM KH_2_PO_4_) or –P (5 μM KH_2_PO_4_). Data are means of four replicates ±SE. Asterisks indicate significant differences between the two P treatments: *P<0.05; ***P<0.001.

Soybean roots were significantly affected by Pi starvation. Compared to P-sufficient control plants, total root length and surface area were 1.8- and 1.9-fold higher, respectively, in the P-deficient treatment (Fig. 2A, B). Primary root lengths did not vary significantly (Fig. 2C), but lateral root lengths nearly doubled in P-deficient plants relative to P-sufficient plants (Fig. 2D). This suggests that the significant increase in total root length caused by P deficiency was mainly due to enhanced growth of lateral roots. Furthermore, it was observed that the root average diameter at the high-P level was higher than that at the low P-level (Fig. S1 at Dryad), suggesting that Pi starvation resulted in thinner soybean roots.

### Effects of Pi starvation on APase activity in soybean roots

**Fig. 3.**
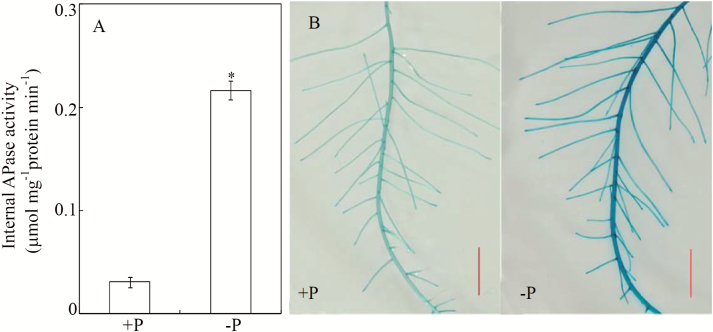
Effects of P availability on the APase activity of soybean roots. (A) Internal APase activity in soybean roots subjected to +P (250 μM KH_2_PO_4_) or –P (5 μM KH_2_PO_4_) conditions. Data are means of four replicates ±SE. Asterisks indicate significant differences between the two P treatments: *P<0.05. (B) APase activity on root surfaces of +P and –P soybean plants detected by BCIP staining. Scale bars are 2 cm.

To further characterize the effects of P deficiency on soybean roots, APase activity was investigated at the two P levels. The results showed that APase activity increased with Pi starvation (Fig. 3). Internal APase activity in P-deficient soybean roots was about six times higher than in the P-sufficient roots (Fig. 3A). Similarly, the root-associated APase activity was also enhanced by Pi starvation, as indicated by the higher intensity of blue color derived from BCIP hydrolysis along root surfaces (Fig. 3B).

### Identification of Pi starvation-responsive CWPs in soybean roots

Cell wall proteins collected from soybean roots subjected to different P treatments were analysed using the iTRAQ technique. In total, 71 proteins were found to be significantly regulated by Pi starvation, including 30 with enhanced and 41 with suppressed accumulation (Tables S1, S2 at Dryad). Among these 71 differentially accumulated proteins, 53 were predicated as secreted and considered as cell wall-associated proteins according to Signal-P V3 and Secretome P 1.0 Server analysis, including 27 up-regulated and 26 down-regulated proteins, which were classified into six groups according to their potential functions (Table 1). The up-regulated CWPs were classified into the functional groups as follows: carbohydrate metabolism accounted for seven proteins, oxido-reduction for five, protein modification and turnover for three, miscellaneous functions for three, and unknown functions for nine. In the miscellaneous group, two purple acid phosphatases were identified, including purple acid phosphatase 1-like (GmPAP1-like) and purple acid phosphatase 22-like (GmPAP22-like), which respectively exhibited 1.7- and 2.3-fold more accumulation in P-deficient roots than in P-sufficient roots (Table 1).

The down-regulated proteins separated into the functional groups as follows: carbohydrate metabolism accounted for eight proteins, oxido-reduction for four, protein modification and turnover for three, signal transduction for one, miscellaneous functions for three, and unknown functions for seven (Table 1).

**Table 1.** iTRAQ identification of proteins regulated by Pi starvation and predicted to be CWPs.

Number	Description	Locus name	Protein accession	Putative function	Fold-change
Up-regulated					
1	Laccase-7-like	Glyma.U027400	XP_003520942	Carbohydrate metabolism	7.0
2	Polygalacturonase PG1 precursor	Glyma.19G006200	NP_001238091	Carbohydrate metabolism	6.9
3	Polygalacturonase inhibitor 2 precursor	Glyma.08G079100	XP_003531065	Carbohydrate metabolism	5.7
4	Probable polygalacturonase isoform X2	Glyma.08G017300	XP_003532926	Carbohydrate metabolism	3.3
5	Probable polygalacturonase	Glyma.09G256100	XP_003533597	Carbohydrate metabolism	1.8
6	Dirigent protein 20-related	Glyma.11G150400	NP_001238114	Carbohydrate metabolism	1.7
7	Dirigent protein 22-like	Glyma.01G127200	XP_003516990	Carbohydrate metabolism	1.7
8	Cationic peroxidase 1-like-1	Glyma.03G038700	XP_003522119	Oxido-reduction	2.4
9	Cationic peroxidase 1-like-2	Glyma.18G211000	XP_003552297	Oxido-reduction	2.2
10	Peroxidase 39-like	Glyma.12G195600	XP_003540317	Oxido-reduction	1.7
11	Reticuline oxidase-like protein-like	Glyma.15G134300	XP_003546286	Oxido-reduction	6.2
12	Peroxidase A2-like	Glyma.15G129200	XP_014623798	Oxido-reduction	4.6
13	Glu S.griseus protease inhibitor	Glyma.20G205800	XP_003556368	Protein modification and turnover	4.3
14	Kunitz trypsin protease inhibitor-like precursor	Glyma.16G211700	NP_001237751	Protein modification and turnover	4.2
15	Aspartic proteinase nepenthesin-1-like	Glyma.11G157000	XP_003538263	Protein modifcation and turnover	1.8
16	Purple acid phosphatase 1-like	Glyma.08G291600	XP_003532035	Miscellaneous protein	1.7
17	Purple acid phosphatase 22-like	Glyma.10G071000	XP_003537064	Miscellaneous protein	2.3
18	Nuclease S1-like	Glyma.01G083300	XP_003516835	Miscellaneous protein	4.9
19	Polyvinylalcohol dehydrogenase-like	Glyma.09G239900	XP_006587758	Unknown	3.5
20	Clathrin light chain 1-like	Glyma.15G072100	XP_003545895	Unknown	2.4
21	AIR12 precursor	Glyma.03G088900	NP_001237825	Unknown	2.1
22	Early nodulin-like protein	Glyma.06G061200	NP_001235764	Unknown	1.8
23	Vacuolar-sorting receptor 1-like isoform 1	Glyma.01G242800	XP_003517598	Unknown	1.6
24	Uncharacterized protein	Glyma.15G131400	XP_014623068	Unknown	4.7
25	Uncharacterized protein	Glyma.09G025200	XP_003534773	Unknown	3
26	Uncharacterized protein	Glyma.02G154700	XP_003520231	Unknown	2.3
27	Uncharacterized protein	Glyma.11G150300	NP_001238117	Unknown	2.0
Down-regulated					
1	Glucan endo-1,3-beta-glucosidase-like	Glyma.18G291500	XP_003552703	Carbohydrate metabolism	0.3
2	Glucan endo-1,3-beta-glucosidase, basic isoform-like	Glyma.15G142400	XP_003546326	Carbohydrate metabolism	0.5
3	Germin-like protein 3	Glyma.16G060900	NP_001236994	Carbohydrate metabolism	0.3
4	Germin-like protein subfamily 1 member 7	Glyma.19G086000	XP_003547666	Carbohydrate metabolism	0.5
5	NADH dehydrogenase	Glyma.20G108500	NP_001235228	Carbohydrate metabolism	0.1
6	Extensin-like protein-like	Glyma.13G178700	XP_003528459	Carbohydrate metabolism	0.3
7	Polygalacturonase inhibitor-like	Glyma.19G145200	XP_003554195	Carbohydrate metabolism	0.7
8	Alpha-xylosidase-like	Glyma.01G081300	XP_003516826	Carbohydrate metabolism	0.5
9	Peroxidase 16-like-1	Glyma.09G057100	XP_003533723	Oxido-reduction	0.4
10	Peroxidase 16-like-2	Glyma.13G098400	XP_003543742	Oxido-reduction	0.5
11	Peroxidase 3-like	Glyma.03G208200	XP_003520731	Oxido-reduction	0.6
12	Copper amino oxidase precursor	Glyma.17G019300	NP_001237211	Oxido-reduction	0.6
13	Serine carboxypeptidase-like	Glyma.09G226700	XP_003534383	Protein modification and turnover	0.2
14	Subtilisin-like protease	Glyma.12G031800	XP_003540860	Protein modification and turnover	0.4
15	Aspartic proteinase nepenthesin-1-like	Glyma.08G321200	XP_003530761	Protein modification and turnover	0.6
16	Agamous-like MADS-box protein AGL8-like	Glyma.13G052100	XP_003544014	Signal transduction	0.4
17	Acyl CoA binding protein	Glyma.09G214500	NP_001237529	Miscellaneous protein	0.6
18	Gamma-glutamyl hydrolase precursor	Glyma.13G267900	NP_001235549	Miscellaneous protein	0.6
19	Bark storage protein A isoform X1	Glyma.07G083900	XP_003528930	Miscellaneous protein	0.6
20	Osmotin-like protein-like	Glyma.12G064300	XP_003539684	Unkown	0.2
21	Programmed cell death protein 5-like	Glyma.08G082100	XP_003524788	Unkown	0.4
22	Polyadenylate-binding protein 2-like	Glyma.02G103900	XP_003518706	Unkown	0.5
23	Protein EXORDIUM-like	Glyma.04G100400	XP_003522788	Unkown	0.6
24	Uncharacterized protein	Glyma.11G048100	NP_001239639	Unkown	0.5
25	Uncharacterized protein	Glyma.10G090100	NP_001239749	Unkown	0.5
26	Uncharacterized protein	Glyma.06G319500	XP_003526379	Unkown	0.6

### Expression patterns of corresponding genes encoding CWPs in response to Pi starvation

**Fig. 4.**
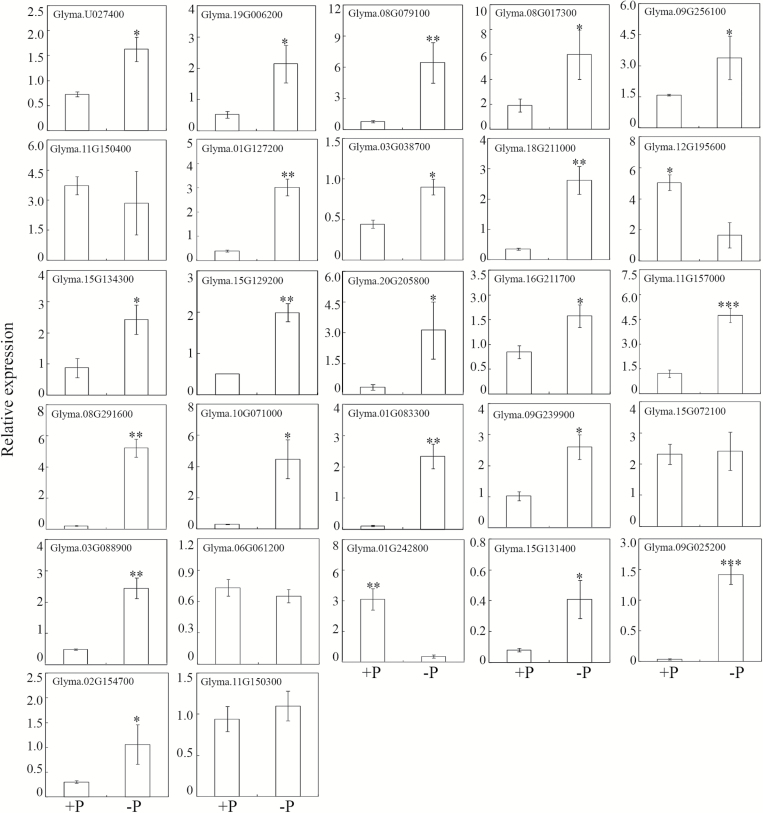
Transcription levels of genes encoding Pi-starvation up-regulated CWPs. qRT-PCR was conducted to analyse gene expression in roots subjected to +P (250 μM KH_2_PO_4_) or –P (5 μM KH_2_PO_4_) treatments. Data are means of four independent replicates ±SE. Asterisks indicate significant differences between the two P treatments: *P<0.05; **P<0.01; ***P<0.001.

**Fig. 5.**
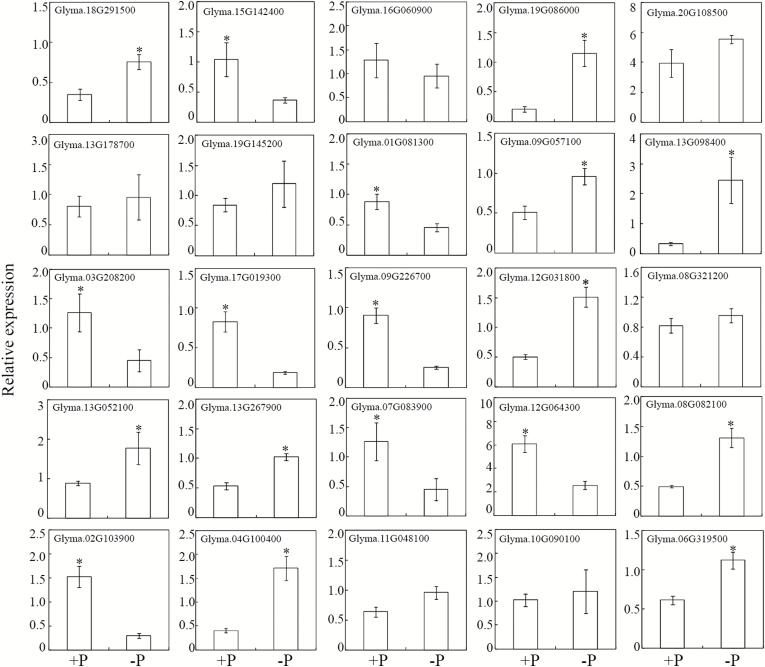
Transcription levels of genes encoding Pi-starvation down-regulated CWPs. qRT-PCR was conducted to analyse gene expression in roots subjected to +P (250 μM KH_2_PO_4_) or –P (5 μM KH_2_PO_4_) treatments. Data are means of four independent replicates ±SE. Asterisks indicate significant differences between the two P treatments: *P<0.05.

The transcriptional levels of genes encoding all the 53 differentially accumulated CWPs were analysed through qRT-PCR, except for the Acyl CoA binding protein (accession number NP_001237529), since no specific PCR primers could be designed to detect its expression. Consistent with the protein accumulation patterns, the transcription levels of 21 genes encoding Pi-starvation up-regulated proteins were significantly increased by P deficiency in soybean roots (Fig. 4). Among them, the transcription of *GmPAP1-like* and *GmPAP22-like* increased by 25- and 9-fold, respectively, in response to P deficiency. Of the remaining up-regulated CWPs, the transcription of four genes was not affected by Pi starvation, and the transcription of two genes was down-regulated (Fig. 4). For down-regulated proteins, the transcription levels of only eight corresponding genes were suppressed, while transcription of seven corresponding genes was not affected, and ten were even up-regulated by Pi starvation (Fig. 5).

### Phylogenetic analysis and expression patterns of GmPAP1-like

**Fig. 6.**
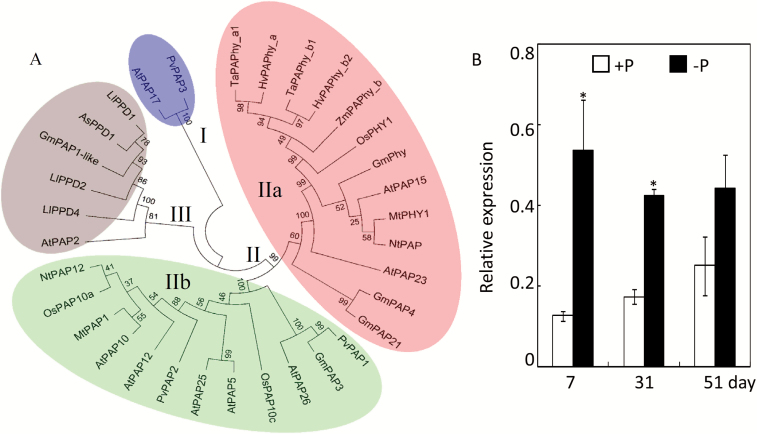
Phylogenetic analysis and expression patterns of *GmPAP1-like*. (A) Phylogenetic analysis of soybean GmPAP1-like and GmPAP22-like (also named as GmPAP21) with other plant PAPs. The first two letters of each PAP protein label represent the abbreviated species name: As, *Astragalus sinicus*; At, *Arabidopsis thaliana*; Gm, *Glycine max*; Hv, *Hordeum vulgare*; Ll, *Lupinus luteus*; Mt, *Medicago truncatula*; Nt, *Nicotiana tabacum*; Os, *Oryza sativa*; Pv, *Phaseolus vulgaris*; Ta, *Triticum aestivum*; Zm, *Zea mays*. The numerals I, II, and III designate the three groups of PAP proteins. Group II was further divided into two subgroups (IIa and IIb). Bootstrap values (%) are indicated for major branches. (B) Expression patterns of soybean *GmPAP1-like* in response to Pi starvation at different growth stages. *P<0.05. (This figure is available in color at *JXB* online.)

A phylogenetic tree of GmPAP1-like, GmPAP22-like, and 32 other PAPs with known functions was constructed based on amino acid sequences. The phylogenetic tree showed that the PAPs could be classified into three distinct clades (I–III; Fig. 6A). Clade I consisted of two PAPs, AtPAP17 and PvPAP3. Clade II was further divided into two subgroups (IIa and IIb), with GmPAP22-like (also reported as GmPAP21) together with other PAPs identified as phytases falling into clade IIa. GmPAP1-like clustered with AtPAP2, three diphosphonucleotide phosphatase/phosphodiesterase (PPD) proteins from white lupin (LjPPD1/2/4), and one PPD from *Astragalus sinicus* (AsPPD1) to form clade III. This clustering indicates that GmPAP1-like belongs to the PPD subfamily of plant PAPs (Fig. 6A).

Since GmPAP22-like has recently been suggested to be involved in soybean nodule growth (Li *et al.*, 2017), GmPAP1-like was further selected and characterized to test for functions in soybean root adaptation to Pi starvation. Expression patterns of *GmPAP1-like* were investigated at different growth stages in soybean roots at the two P levels. The results showed that expression increased significantly in response to Pi starvation in both seedlings and flowering plants (Fig. 6B), as indicated by 5- and 2.5-fold increases in transcription, respectively, in –P compared to +P treatments. In contrast, there was no significant difference for *GmPAP1-like* expression between the two P treatments at the maturity stage. In addition, the expression of *GmPAP1-like* in response to Pi starvation was also investigated in nine other soybean genotypes. After 10 d of low-P treatment the transcripts of *GmPAP1-like* in the roots of all the genotypes were markedly up-regulated by Pi starvation (Fig S2 at Dryad). In contrast, no genotypic differences were observed under P-deficient conditions.

### Contribution of cell wall-localized GmPAP1-like to the enhanced APase activity in transgenic bean hairy roots

**Fig. 7.**
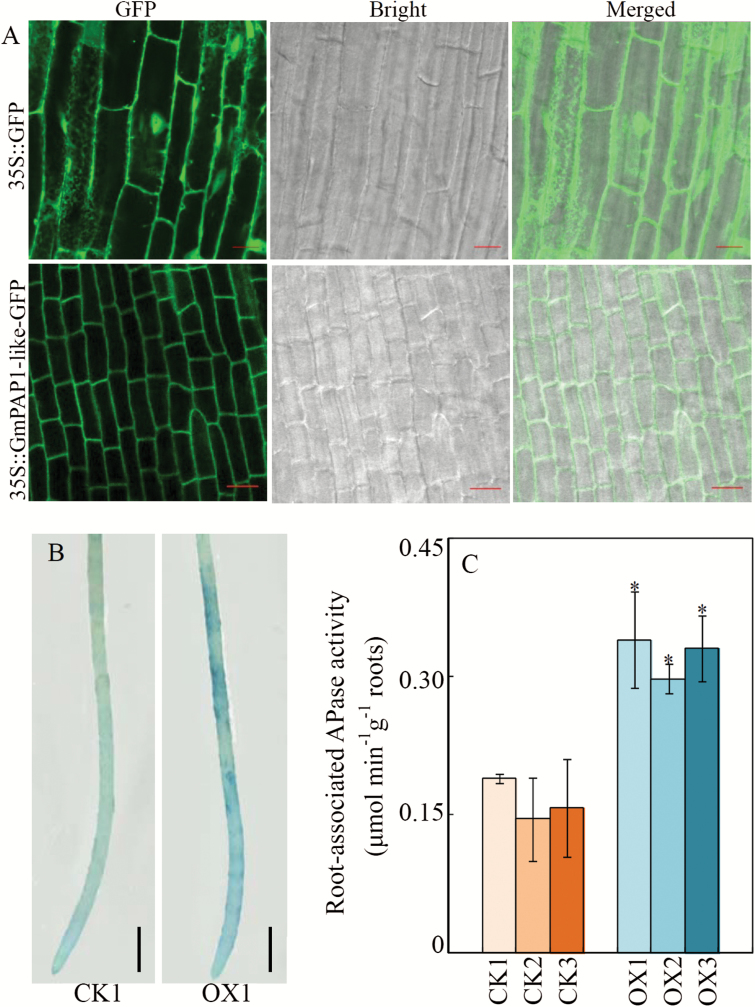
Subcellular localization of GmPAP1-like and root-associated APase activity in transgenic bean hairy roots overexpressing *GmPAP1-like*. (A) Bean hairy roots expressing *35S::GFP* (top row) and *35S:: GmPAP1-like-GFP* (bottom row) were observed by confocal laser scanning microscopy. Scale bars are 20 μm; (B) Root-associated APase activity of bean hairy roots transformed with the empty vector (CK1) or *GmPAP1-like* (OX1) detected by BCIP staining. Scale bars are 5 mm. (C) Root-associated APase activity of transgenic bean hairy roots. CK1, CK2, and CK3 represent three transgenic hairy root lines transformed with the empty vector; OX1, OX2, and OX3 indicate three transgenic hairy root lines with *GmPAP1-like* overexpression. Data are means of four biological replicates ±SE. Asterisks indicate significant differences between *GmPAP1-like* overexpression lines and CK lines: *P<0.05.

To further test whether GmPAP1-like is a cell wall-localized protein, *GmPAP1-like* was fused with *GFP* and overexpressed in bean hairy roots. Compared with the nuclear and cytoplasmic localization of GFP in the empty-vector control, the green fluorescence of GmPAP1-like-GFP was predominantly detected on the cell periphery (Fig. 7A; Fig. S3 at Dryad), and it merged with the red fluorescence derived from PI staining, strongly suggesting that GmPAP1-like is localized in cell walls.

Root-associated APase activity was also determined in bean hairy roots with *GmPAP1-like* overexpression *in vivo*. The results showed that *GmPAP1-like* overexpression significantly enhanced root-associated APase activity, as indicated by the development of darker BCIP staining on the surfaces of *GmPAP1-like* over-expressing hairy roots (Fig. 7B). Consistent with the staining, root-associated APase activity measured using ρ-NPP as the substrate was 2.1-, 1.8-, and 2.0-fold higher for three *GmPAP1-like* over-expression lines compared with the empty-vector controls (Fig. 7C).

### Overexpression of GmPAP1-like enhances extracellular dNTP utilization

**Fig. 8.**
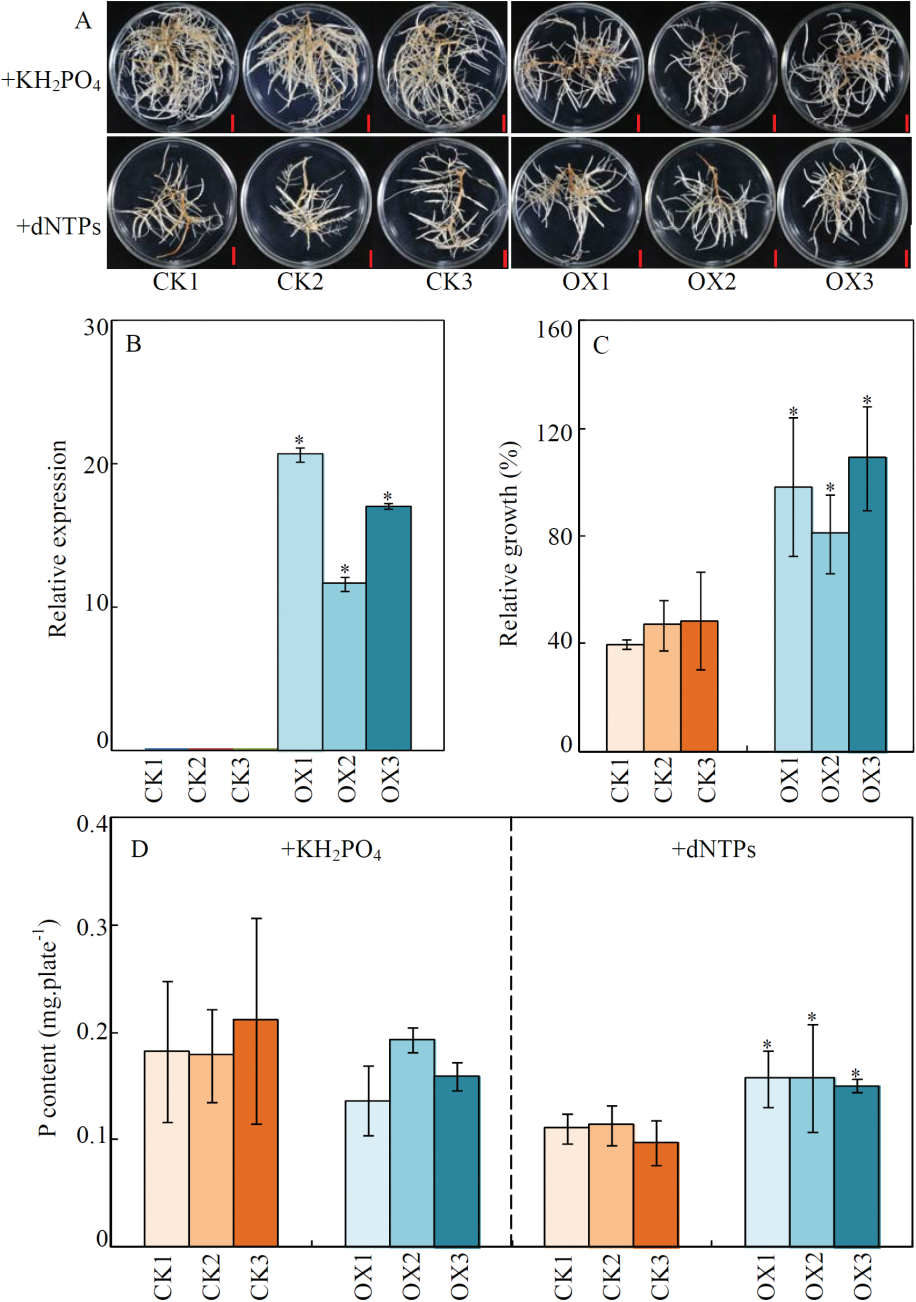
Growth and P content of transgenic bean hairy roots supplied with different P sources. (A) Phenotypes of transgenic bean hairy roots supplied with different P sources. Scale bars are 1 cm. (B) Expression levels of *GmPAP1-like* in transgenic been hairy roots. (C) Relative growth of transgenic bean hairy roots. Relative growth (%) = 100×(Increase of fresh weight in dNTP / Increase of fresh weight in KH_2_PO_4_). (D) P content in transgenic bean hairy roots. Uniform transgenic bean hairy roots were grown on MS medium supplied with 1.2 mM KH_2_PO_4_ or 0.4 mM dNTP for 14 d. Fresh weight and P content were measured, and the fresh weight was further used for the calculation of relative growth of the roots. CK1, CK2, and CK3 are three transgenic lines transformed with an empty vector; OX1, OX2, and OX3 represent transgenic lines with *GmPAP1-like* overexpression. Data are means of four biological replicates ±SE. Asterisks indicate significant differences between *GmPAP1-like* overexpression lines and CK lines: *P<0.05.

To investigate the potential function of *GmPAP1-like* in relation to organic-P utilization, transgenic bean hairy roots overexpressing *GmPAP1-like* were generated and confirmed by qRT-PCR (Fig. 8B). Uniform transgenic lines were then supplied with one of two P sources, either KH_2_PO_4_ or dNTP, for 2 weeks. The results showed that when dNTP was supplied as the sole P source, the fresh weight of control lines (CK) was significantly lower than that observed when KH_2_PO_4_ was the P source (Fig. 8A; Fig. S4 at Dryad). The fresh weight of all three *GmPAP1-like* overexpression lines (OX) did not vary between KH_2_PO_4_ and dNTP treatments, which thereby resulted in a higher relative growth with dNTP for the OX lines than for the CK lines (Fig. 8A, C; Fig. S4 at Dryad).

Consistent with the observed effects of P source on relative growth, P contents in the dNTP treatment were 46%, 47%, and 40% higher in the three OX lines compared with the CK lines (Fig. 8D). On the other hand, the P content was similar between the OX and CK lines when KH_2_PO_4_ was supplied as the sole source of P (Fig. 8D). These results suggest that GmPAP1-like participates in extracellular dNTP utilization in soybean.

## Discussion

Low Pi availability is one of the primary factors limiting plant growth on many soils. Over time, plants have evolved many adaptations to maintain growth in low-P soils, including alteration of root morphology and increased exudation of APase, both of which might involve modifications in root cell walls (Cassab and Varner, 1988; Kaplan and Hagemann, 1991; Chiou and Lin, 2011; Liang *et al.*, 2014; Sun *et al.*, 2016*a*). Cell wall modifications are continuously controlled by the enzymatic actions of CWPs, which account for about 10% of the cell wall dry weight (Cassab, 1998). It is therefore important to study the regulation of CWP accumulation by low-P stress, as well as to determine how this might underlie the physiological and morphological adaptations of plant roots to Pi starvation.

Various methods have been applied to extract CWPs, which can be loosely or tightly bound to the cell wall matrix in higher plants, green alga, and fungi (Fry, 1988; Pardo *et al.*, 2000; Chivasa *et al.*, 2002; Wang *et al.*, 2004; Watson *et al.*, 2004; Feiz *et al.*, 2006; Jung *et al.*, 2008; Calderan-Rodrigues *et al.*, 2014). Among the various methodologies, those developed by Feiz *et al.* (2006) are considered to be optimum for the extraction of water-soluble and weakly bound plant CWPs, with yields that include up to 73% of the proteins identified as potential CWPs (Feiz *et al.*, 2006). Following these methods, CWPs were extracted from both P-sufficient and P-deficient soybean roots and yielded a total of 71 proteins regulated by Pi starvation (Tables S1 and S2 at Dryad). Moreover, 53 of the 71 differentially expressed proteins (74.6%) were implicated as potential CWPs by SignalP and Secretome analyses (Table 1). The identification of these root CWPs with differential accumulation strongly suggests the presence of complex strategies for remodeling root cell walls in adaptive responses to P deficiency.

The CWPs with differential accumulation in response to P deficiency fell into six functional groups, namely carbohydrate metabolism, oxido-reduction, protein modification and turnover, miscellaneous proteins, signal transduction, and unknown (Table 1). Among the proteins in the carbohydrate metabolism category, three polygalacturonases were found to be up-regulated. Polygalacturonase orthologues have been reported to function as pectin-digesting enzymes in a wide range of developmental processes, such as cell separation, fruit ripening, and cell growth in many plant species (Hadfield and Bennett, 1998; Torki *et al.*, 1999; Daher and Braybrook, 2015). A recent study showed that overexpressing a *polygalacturonase* gene, *PGX1*, resulted in enhanced hypocotyl elongation (Xiao *et al.*, 2014). On the other hand, mutation of an *exo-polygalacturonase* gene, *NIMNA*, caused cell elongation defects in the early embryo, and markedly reduced suspensor length in Arabidopsis (Babu *et al.*, 2013). Hence we suggest that the polygalacturonases identified in the present study might play similar roles in cell wall loosening, thus enabling the enhanced elongation of soybean roots under P-deficiency (Fig. 2). Other proteins in the carbohydrate metabolism category, such as dirigent-like protein, beta-glucosidase, and extensin-like protein, have been well documented as functioning in the biosynthesis and differentiation of cell walls in other plant species (Davin and Lewis, 2000; Baiya *et al.*, 2014; Draeger *et al.*, 2015). Placing our current results in the context of previous reports suggests that soybean root cell walls undergo complex modifications mediated through changes in abundance of CWPs in response to low-P stress.

Another large group of P-responsive CWPs appears to participate in the metabolism of reactive oxygen species (ROS) (Table 1). The dynamics of ROS in plant cell walls has been considered to play direct roles in cell wall loosening via polysaccharide cleavage (Fry, 1998; Fry *et al.*, 2001; Liszkay *et al.*, 2003), thereby affecting cell extension and growth (Rodriguez *et al.*, 2002; Liszkay *et al.*, 2004; Chacón-López *et al.*, 2011). Furthermore, ROS production has recently been reported to be spatially and temporally regulated by Pi starvation in Arabidopsis, which subsequently affects the meristem exhaustion process triggered by Pi-starvation (Tyburski *et al.*, 2009; Chacón-López *et al.*, 2011). Hence the proteins identified in this study as functioning in oxido-reduction might be involved in the processes of cell wall stiffening/loosening through precise regulation of local ROS concentrations, which thus regulates soybean root growth under P-deficient conditions (Fig. 2).

The CWPs identified as functioning in signal transduction, protein modification and turnover, miscellaneous functioning, and unknown functions further reflect the complexity of cell wall modifications associated with soybean adaptation to low-P stress. The agamous-like MADS-box proteins, for example, have been reported to be involved in root development and nutrient-deficiency responses in a wide range of plants, including Arabidopsis, rice and orange (Zhang and Forde, 1998; Alvarez-Buylla *et al.*, 2000; Burgeff *et al.*, 2002; Yu *et al.*, 2014; Sun *et al.*, 2016*b*). Moreover, subtilisin-like proteases have also been suggested to function in the modulation of plant morphology in Arabidopsis, with reported roles in epidermal development for ALE1 (Tanaka *et al.*, 2001), lateral root formation for AIR3 (Neuteboom *et al.*, 1999), and xylem development for XSP1 (Zhao *et al.*, 2000). In the current study, therefore, the subtilisin-like protease and agamous-like MADS-box protein identified as P-responsive CWPs might have roles in regulating soybean root development and growth in response to P deficiency. Other proteins, such as acyl-CoA-binding protein and EXORDIUM-like, might also be associated with development and phytohormone-regulated root growth, respectively (Coll-Garcia *et al.*, 2004; Du *et al.*, 2016). However, elucidation of their exact functions in soybean root responses to Pi starvation will require further investigation.

We found that soybean root-associated APase activity significantly increased with Pi starvation (Fig. 3). Further analysis identified two PAPs, GmPAP22-like and GmPAP1-like, in the CWP fraction with enhanced transcription and protein accumulation in response to low-P stress (Table1, Fig. 4). It is well documented that increased root-associated APase activity in response to Pi-starvation is conserved among diverse plant species (Asmar *et al.*, 1995; George *et al.*, 2008; Tian and Liao, 2015). These responses have been correlated with increased transcription and post-transcriptional modification of PAPs (Robinson *et al.*, 2012; Wang *et al.*, 2014; Liu *et al.*, 2016). Placing our current expression and APase activity results in the context of previous reports, it is reasonable to suggest that GmPAP22-like and GmPAP1-like contribute significantly to the increased root-associated APase activity observed in soybean in response to low-P stress.

GmPAP22-like, which is also named GmPAP21, has been suggested to function in P recycling and metabolism in soybean (Li *et al.*, 2012, 2017). In contrast, little information is available on the functional characterization of GmPAP1-like in soybean, and hence it was chosen for more detailed analysis. The phylogenetic tree showed that GmPAP1-like had high similarity and clustered with PPDs, such as AsPPD1 in *Astragalus sinicus* and LlPPD1 in *Lupinus luteus* (Fig. 6A). The PPD proteins have been characterized as a subfamily of PAPs, with differences from typical PAPs in certain features (Olczak *et al.*, 2000, 2009).

The limited information available suggests that the PPDs identified to date have divergent functions and are not directly involved in plant adaptations to P deficiency. For example, AsPPD1 from *A. sinicus* plays a role in controlling the symbiotic ADP levels (Wang *et al.*, 2015), while AtPAP2 in Arabidopsis has been found to modulate carbon metabolism in plastids and mitochondria (Sun *et al.*, 2012; Zhang *et al.*, 2012). In the present study, it was found that *GmPAP1-like* overexpression in bean hairy roots significantly increased dNTP utilization efficiency, as indicated by higher P content and relative growth of overexpressing lines compared to control lines (Fig. 8C, D). Utilization of nucleic acid P has been reported for a variety of plant PAPs, including PvPAP1 in bean, OsPAP10a in rice, AtPAP12 and AtPAP26 in Arabidopsis, and SgPAP7, SgPAP10, and SgPAP26 in stylo (Liang *et al.*, 2012; Robinson *et al.*, 2012; Tian *et al.*, 2012; Tian and Liao, 2015; Liu *et al.*, 2016). Therefore, GmPAP1-like might play a role in soybean P nutrition by facilitating Pi acquisition from organic P sources, which is similar in functionality to typical PAPs. However, the fresh weights of *GmPAP1-like* overexpression bean hairy roots were lower than those of control lines under normal conditions (Fig. S4 at Dryad). This is consistent with the observation that overexpression of *GmPAP21* inhibits growth in soybean (Li *et al.*, 2017). Taken together, the results suggest that PAP might also affect internal P-derivative metabolism, and thus influences plant growth, which merits further investigation for other roles of GmPAP1-like.

In summary, it is well known that P efficiency in plants is largely determined by root morphology and architecture, which are affected by root cell wall modification and metabolism that rely on the enzymatic activities of CWPs. However, there is a lack of proteomics data available for CWP responses to P deficiency, which is vital to further understand molecular mechanisms underlying root adaptations to P deficiency. To the best of our knowledge, this is the first study to identify CWPs responsive to Pi starvation in plants through proteomics analysis. Our results strongly suggest that CWPs with diverse functions participate in complex modifications of root cell walls in soybean responses to P deficiency. Among the CWPs that have been identified, a cell wall-localized purple acid phosphatase, GmPAP1-like, contributes to the enhanced root-associated APase activity in response to Pi starvation and is involved in the acquisition of Pi from extracellular dNTPs.

## Data deposition

The following data are available at Dryad Data Repository: http://dx.doi.org/10.5061/dryad.6t1f5.

Dataset 1. Mass spectrometry/spectra information for the iTRAQ analysis of soybean cell wall proteins.

Table S1. General information for the 41 proteins with abundance down-regulated in response to low-P stress. 

Table S2. General information for the 30 proteins with abundance up-regulated in response to low-P stress.

Table S3. Primer pairs for qRT-PCR analysis of corresponding genes responsive to Pi starvation.

Fig. S1. Phenotype and average root diameter of soybean subjected to P-sufficient and P-deficient conditions. 

Fig. S2. Expression patterns of *GmPAP1-like* in response to Pi starvation among different soybean genotypes. 

Fig. S3. Subcellular localization of GmPAP1-like in transgenic bean hairy roots overexpressing *GmPAP1-like*.

Fig. S4. Effects of overexpression of *GmPAP1-like* on the growth of transgenic bean hairy roots.
